# Supplementary material for: Monitoring fatty acid trafficking during Drosophila melanogaster oogenesis reveals a role for the triglyceride synthase DGAT1 in protecting mitochondrial integrity
Source: bioRxiv. 2026 Aug 12:2026.08.10.743872. Preprint. [Version 1] doi: 10.64898/2026.08.10.743872 (PMC13640666; doi:10.64898/2026.08.10.743872)
Supplement: Supplementary file 1 [file NIHPP2026.08.10.743872v1-supplement-1.pdf]

bioRxiv preprint doi: https://doi.org/10.64898/2026.08.10.743872; this version posted August 12, 2026. The copyright holder for this preprint (which was not certified by peer review) is the author/funder, who has granted bioRxiv a license to display the preprint in perpetuity. It is made available under a CC-BY 4.0 International license.

Supporting Information

S1 Fig

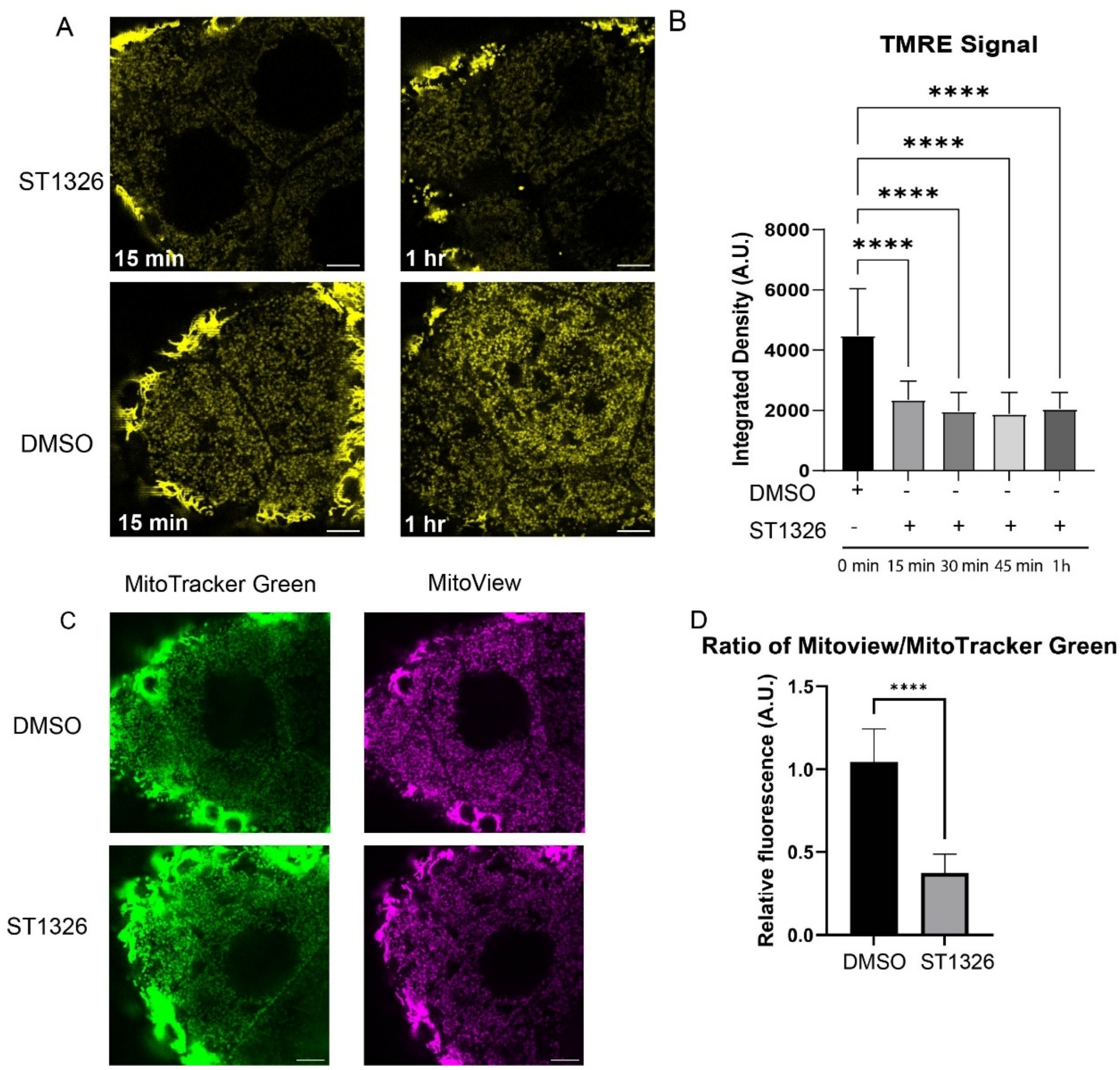

bioRxiv preprint doi: https://doi.org/10.64898/2026.08.10.743872; this version posted August 12, 2026. The copyright holder for this preprint (which was not certified by peer review) is the author/funder, who has granted bioRxiv a license to display the preprint in perpetuity. It is made available under a CC-BY 4.0 International license.

S1 Fig. **Pharmacological Inhibition of CPT1 Reduces Membrane Potential.** A) Live images of wild-type S9 follicles treated with ST1326 or DMSO vehicle control, captured at 15 minutes and 1 hour post-treatment. Follicles were stained with TMRE (25 nM) to assess mitochondrial membrane potential. B) Quantitation of TMRE integrated density over time in ST1326- and DMSO-treated S9 follicles. ST1326 treatment causes a significant reduction in TMRE signal relative to DMSO controls at all timepoints examined. C) Wild-type S9 follicles stained with MitoTracker Green (mitochondrial mass marker) and MitoView (mitochondrial membrane potential dye) following DMSO or ST1326 treatment. D) Quantitation of MitoView signal normalized to MitoTracker Green signal in S9 nurse cells, showing a significant reduction in membrane potential per unit mitochondrial mass in ST1326-treated follicles relative to DMSO controls. Data represents SD. ****$p < 0.0001$. Scale bars: 10 μm (A, C).

bioRxiv preprint doi: https://doi.org/10.64898/2026.08.10.743872; this version posted August 12, 2026. The copyright holder for this preprint (which was not certified by peer review) is the author/funder, who has granted bioRxiv a license to display the preprint in perpetuity. It is made available under a CC-BY 4.0 International license.

S2 Fig

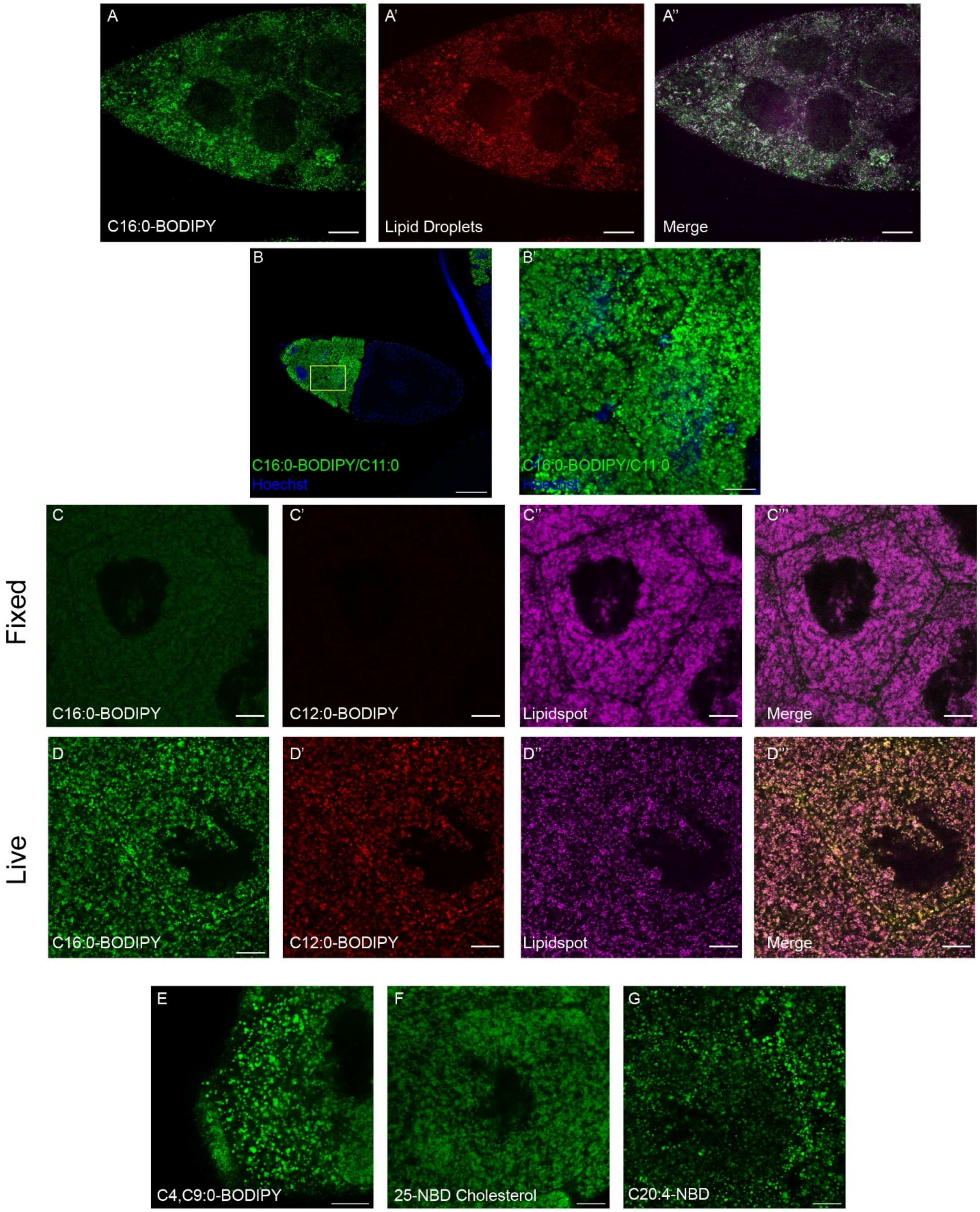

S2 Fig. **Visualizing Sequestration of Fluorescent Fatty Acid by NC LDs.** A-A'') Live images of wild-type S9 follicle from adult female that had been feed C16:0-BODIPY yeast paste for 20

bioRxiv preprint doi: https://doi.org/10.64898/2026.08.10.743872; this version posted August 12, 2026. The copyright holder for this preprint (which was not certified by peer review) is the author/funder, who has granted bioRxiv a license to display the preprint in perpetuity. It is made available under a CC-BY 4.0 International license.

hours. BODIPY signal (A), LDs detected by confocal reflection microscopy (CRM) (A’), merge of the two channels. B) Wild-type follicle supplemented with C16:0-BODIPY/C11:0 (DAG) supplemented WT follicle and stained with Hoechst. B’) Magnified region of the follicle from B. C-D’’) Wild-type S9 nurse cells exposed to C16:0-BODIPY (C, D) and C12:0-BODIPY (C’, D’) and stained with LipidSpot (C’’, D’’). C’’’ and D’’’ show merge of all channels. The follicle in (C-C’’’) was fixed prior to supplementation with fluorescent FAs. The follicle in D-D’’’ was alive during incubation with fluorescent FAs. E) Wild-type nurse cell after incubation with C4:0-BODIPY, C9:0-BODIPY. F) Wild-type nurse cell after incubation with 25-NBD cholesterol. G) Wild-type nurse cell after incubation with C20:4-NBD. Scale Bar A-A’’ = 20μm. Scale Bar B and B’ = 50μm. and 10μm respectively Scale Bar C-G = 10μm.

bioRxiv preprint doi: https://doi.org/10.64898/2026.08.10.743872; this version posted August 12, 2026. The copyright holder for this preprint (which was not certified by peer review) is the author/funder, who has granted bioRxiv a license to display the preprint in perpetuity. It is made available under a CC-BY 4.0 International license.

1086 **S3 Fig**

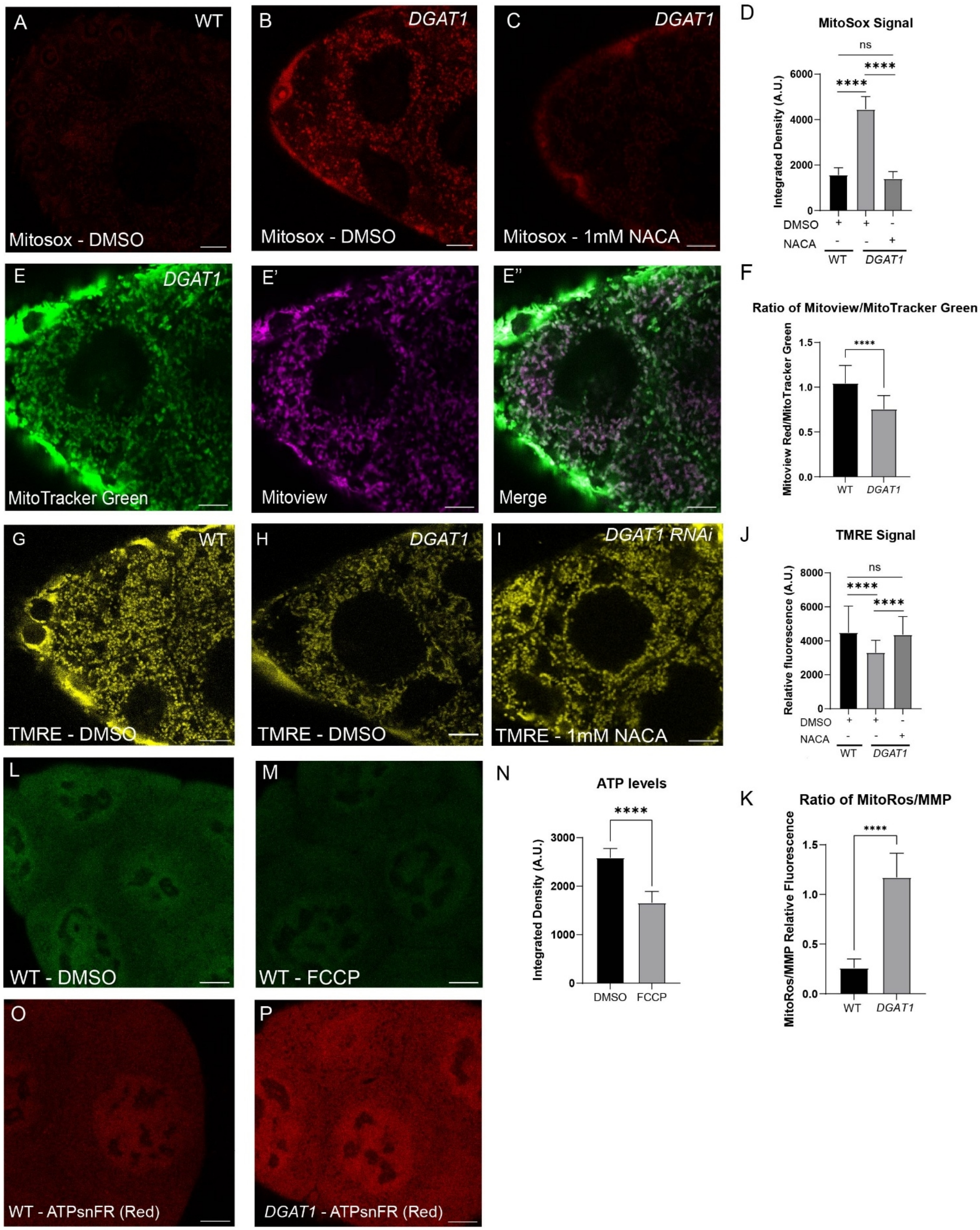

1087

bioRxiv preprint doi: https://doi.org/10.64898/2026.08.10.743872; this version posted August 12, 2026. The copyright holder for this preprint (which was not certified by peer review) is the author/funder, who has granted bioRxiv a license to display the preprint in perpetuity. It is made available under a CC-BY 4.0 International license.

S3 Fig. ***DGAT1* Mutants Display Multiple Mitochondrial Defects.** A) Live image of MitoSox staining for wild-type, B) *DGAT1*, and C) NACA-treated *DGAT1* mutant S9 follicles. D) Quantitation of MitoSox signal for the indicated conditions. E-E'') Live images of the same *DGAT1* follicle stained with MitoTracker Green (E) and MitoView (E'). E" shows merge of both channels. F) Quantitation of the ratio of MitoView and MitoTracker Green relative fluorescence for the indicated genotypes. G-I) Live images of TMRE (25nM) staining for wild-type (G), *DGAT1* (H), and NACA-treated *DGAT1* RNAi (I) S9 follicles. J) Quantitation of TMRE signa for the indicated conditions. K) Quantitation of the ratio of MitoRos to mitochondrial membrane potential (MitoRos/MMP) for wild-type and *DGAT1* mutant S9 follicles. L, M) Live images of ATP synthase sniffer (ATPsnFR) signal in nurse cells of wild-type S9 follicles, untreated (L) or treated with FCCP. N) Quantitation of ATP synthase sniffer signal. O, P) Red channel of ATPsnFR in wild-type and *DGAT1* nurse cells used to normalize green channel signal. Scale Bar A–C, E–E", G–I, L–M, O–P = 10µm.

bioRxiv preprint doi: https://doi.org/10.64898/2026.08.10.743872; this version posted August 12, 2026. The copyright holder for this preprint (which was not certified by peer review) is the author/funder, who has granted bioRxiv a license to display the preprint in perpetuity. It is made available under a CC-BY 4.0 International license.

1114 **S4 Fig**

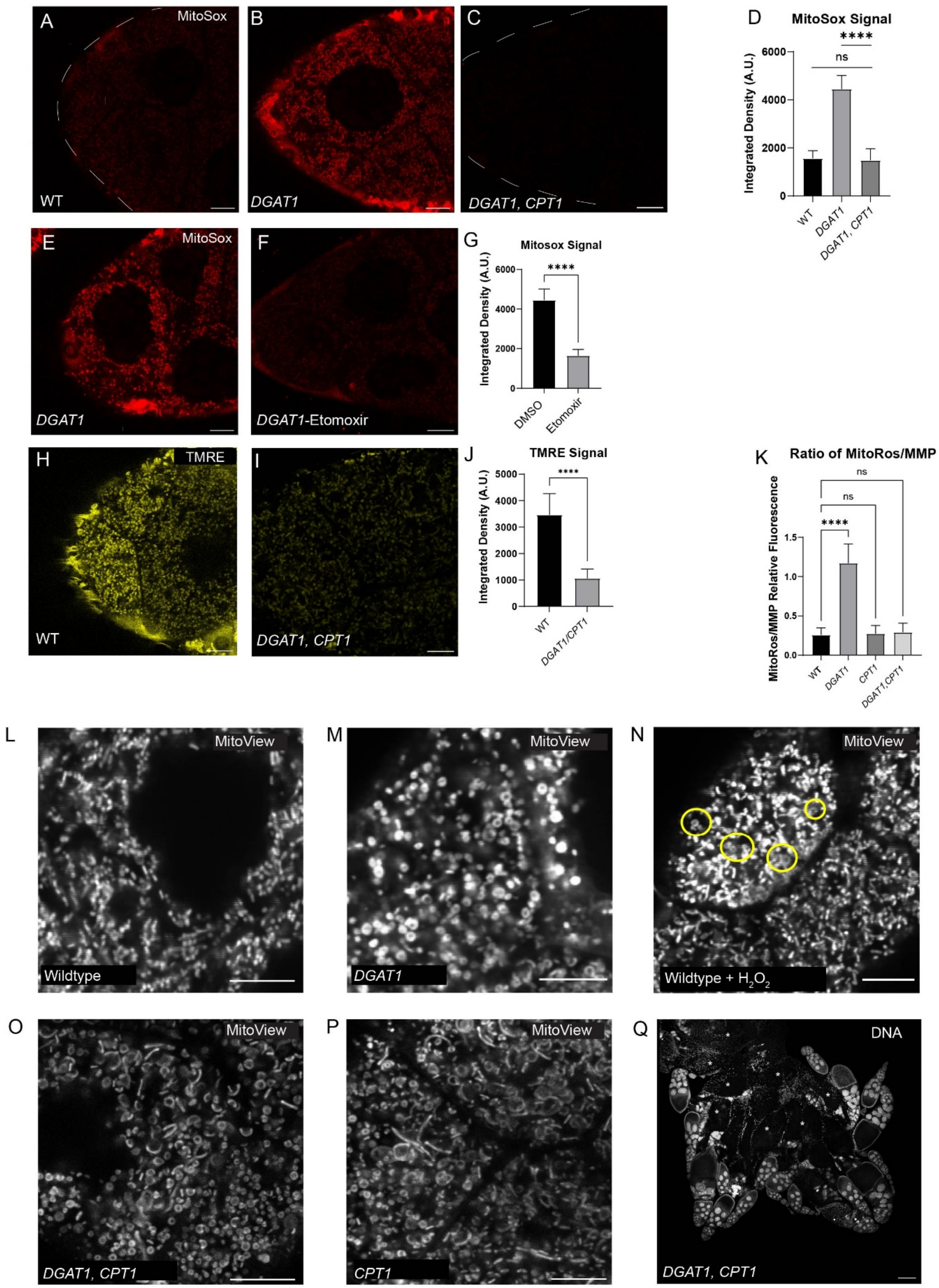

1115

bioRxiv preprint doi: https://doi.org/10.64898/2026.08.10.743872; this version posted August 12, 2026. The copyright holder for this preprint (which was not certified by peer review) is the author/funder, who has granted bioRxiv a license to display the preprint in perpetuity. It is made available under a CC-BY 4.0 International license.

S4 Fig. **Perturbations to DGAT and CPT1 Function Affect both Mitochondrial Bioenergetics and Morphology.** A–C) Representative confocal images of MitoSox staining in wild-type (A) , *DGAT1* mutant (B), and *DGAT1 CPT1* double mutant S9 follicles (C). D) Quantitation of MitoSox integrated density in NCs of S9 follicles across genotypes. *DGAT1* mutants show a significant elevation in MitoSox signal relative to wild type, while signal in *DGAT1 CPT1* double mutants is not significantly different from *DGAT1* single mutants. E–F) Representative confocal images of MitoSox staining in *DGAT1* mutant S9 follicles treated with DMSO vehicle (E) or Etomoxir (F). G) Quantitation of MitoSox integrated density, demonstrating significant reduction of ROS signal following Etomoxir treatment in *DGAT1* mutants. H–I) Representative confocal images of TMRE staining in wild-type (H) and *DGAT1, CPT1* double mutant (I) follicles. J) Quantitation of TMRE integrated density in S9 follicles, demonstrating significantly reduced mitochondrial membrane potential in *DGAT1 CPT1* double mutants relative to wild type. K) Quantitation of the ratio of MitoROS signal to mitochondrial membrane potential (MitoROS/MMP) in S9 follicles across genotypes. L–P) Representative confocal images of MitoView staining in nurse cells from wild-type (L), *DGAT1* mutant (M), wild-type treated with H_2O_2 (yellow circles indicate fragmented mitochondria) (N), *DGAT1 CPT1* double mutant (O), and *CPT1* mutant (P) S9 follicles. Q) Hoechst staining of a *DGAT1 CPT1* whole ovary. Data represent mean ± SD. ****$p < 0.0001$; ns, not significant. Images were acquired on Leica Stellaris (20x) for panel Q only. Scale bars: 10 μm (A–C, E–F, H–I, L–P).

bioRxiv preprint doi: https://doi.org/10.64898/2026.08.10.743872; this version posted August 12, 2026. The copyright holder for this preprint (which was not certified by peer review) is the author/funder, who has granted bioRxiv a license to display the preprint in perpetuity. It is made available under a CC-BY 4.0 International license.

S5 Fig

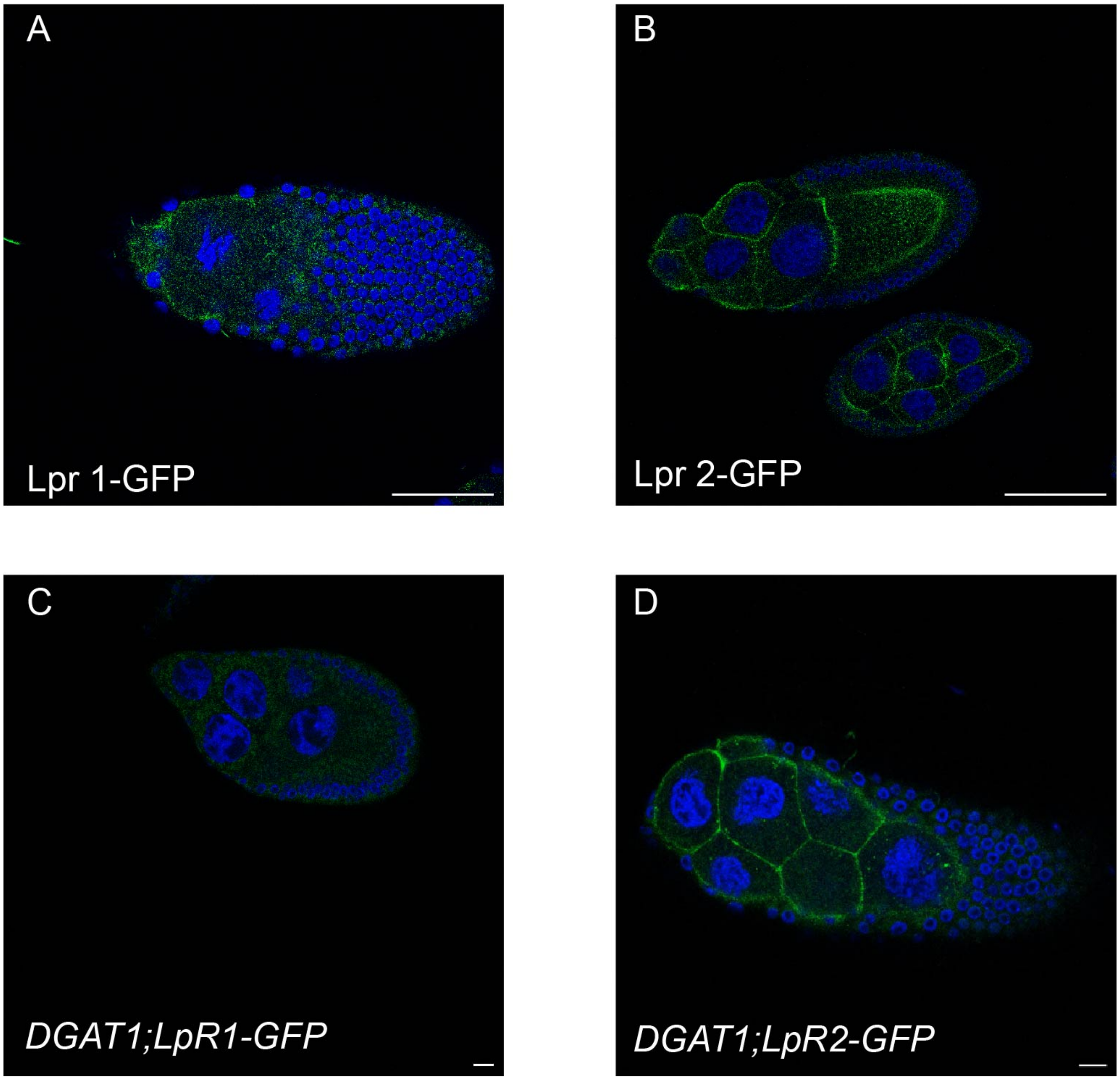

S5 Fig. **Visualizing LpR Expression during Mid-oogenesis.** LpR1- or Lpr2-GFP expressing S9 follicles stained with Hoechst (blue) to detect nuclei. **A**) LpR1-GFP S9 follicle. B) LpR2-GFP S9 follicle. C) *DGAT1*; LpR1-GFP S9 follicle. D) *DGAT1*;LpR2-GFP S9 follicle. Scale Bar A-B = 50µm C-D = 10µm.

bioRxiv preprint doi: https://doi.org/10.64898/2026.08.10.743872; this version posted August 12, 2026. The copyright holder for this preprint (which was not certified by peer review) is the author/funder, who has granted bioRxiv a license to display the preprint in perpetuity. It is made available under a CC-BY 4.0 International license.

Supplemental Movies

S1 Video. Real-Time Visualization of C16:0-BODIPY incubation. **A)** Image sequence of wild-type S10 follicle in maturation media supplemented with 5 μM C16:0-BODIPY (green). **B)** Image sequence of wild-type follicle in maturation media supplemented with 5 μM C16:0-BODIPY (green). CRM was utilized to visualize LD (magenta). Frame rate: 250 frames, 17.86 sec. Total duration: 58 minutes. Scale bar = 50 μm

S2 Video. Real-Time Visualization of C12:0-BODIPY incubation in PDI-GFP S10 follicles. **A)** Image sequence of PDI-GFP nurse cells in maturation media supplemented with 5 μM C12:0-BODIPY. **B)** CRM was used to visualize LDs. **C)** PDI-GFP expression. **D)** Merge of C12:0-BODIPY and CRM. The follicle was not treated with any drug prior to imaging. Frame rate: 16 frames, 4 sec. Total duration: 40 minutes. scale bar 10 μm

S3 Video. Colchicine Treatment of C12:0-BODIPY-Supplemented PDI-GFP S10 Follicle. **A)** Prior to imaging, the follicle was treated with 80μg/mL colchicine for 1 hour in maturation media. PDI-GFP follicle was then supplemented with 5 μM C12:0-BODIPY in maturation media. **B)** CRM was used to visualize LDs. **C)** PDI-GFP expression. **D)** Merge of C12:0-BODIPY and CRM. Frame rate: 15 frames, 3.75 sec. Total duration: 37.5 minutes. Scale bar 10 μm

bioRxiv preprint doi: https://doi.org/10.64898/2026.08.10.743872; this version posted August 12, 2026. The copyright holder for this preprint (which was not certified by peer review) is the author/funder, who has granted bioRxiv a license to display the preprint in perpetuity. It is made available under a CC-BY 4.0 International license.

S1 Table. Fluorescent lipid species

Lipid Species	Company	Saturation
BODIPY™ 500/510 C4, C9 (5-Butyl-4,4-Difluoro-4-Bora-3a,4a-Diaza-s-Indacene-3-Nonanoic Acid))	Thermo Fisher Scientific	Saturated
BODIPY™ FL C12 (4,4-Difluoro-5,7-Dimethyl-4-Bora-3a,4a-Diaza-s-Indacene-3-Dodecanoic Acid)<br>BODIPY™ FL C16 (4,4-Difluoro-5,7-Dimethyl-4-Bora-3a,4a-Diaza-s-Indacene-3-Hexadecanoic Acid)	Thermo Fisher Scientific	Saturated
1-palmitoyl-2-(dipyrrometheneboron difluoride) undecanoyl-sn-glycerol	Avanti Polar Lipids	Saturated
25-[N-[(7-nitro-2-1,3-benzoxadiazol-4-yl)methyl]amino]-27-norcholesterol	Avanti Polar Lipids	N/A
20-[(7-nitro-2-1,3-benzoxadiazol-4-yl)amino] arachidonic acid	Avanti Polar Lipids	Polyunsaturated
